# Protease-Dead Separase Is Dominant Negative in the *C. elegans* Embryo

**DOI:** 10.1371/journal.pone.0108188

**Published:** 2014-09-22

**Authors:** Diana M. Mitchell, Lindsey R. Uehlein-Klebanow, Joshua N. Bembenek

**Affiliations:** Department of Biochemistry, Cellular and Molecular Biology, University of Tennessee, Knoxville, Tennessee, United States of America; Brown University/Harvard, United States of America

## Abstract

Separase is a protease that promotes chromosome segregation at anaphase by cleaving cohesin. Several non-proteolytic functions of separase have been identified in other organisms. We created a transgenic *C. elegans* line that expresses protease-dead separase in embryos to further characterize separase function. We find that expression of protease-dead separase is dominant-negative in *C. elegans* embryos, not previously reported in other systems. The *C. elegans* embryo is an ideal system to study developmental processes in a genetically tractable system. However, a major limitation is the lack of an inducible gene expression system for the embryo. We have developed two methods that allow for the propagation of lines carrying dominant-negative transgenes and have applied them to characterize expression of protease-dead separase in embryos. Using these methods, we show that protease-dead separase causes embryo lethality, and that protease-dead separase cannot rescue separase mutants. These data suggest that protease-dead separase interferes with endogenous separase function, possibly by binding substrates and protecting them from cleavage.

## Introduction

Separase is a cysteine protease with multiple roles during cell division. In a number of these roles the protease activity of separase is required, including cohesin cleavage at the onset of anaphase [1]–[4], DNA repair [5], resolution of chiasmata in mouse oocytes [6], mitotic spindle elongation [7], and centriole duplication [8]–[10]. Additional non-proteolytic functions of separase have been identified, including anaphase exit [11] and Cdc14 early anaphase release (FEAR) pathway activation [12], [13], and polar body extrusion in mouse oocytes [6]. Importantly, these studies examined protease-dead separase in separase mutant cells and concluded that separase can promote signaling events independent of its protease activity. There have been no studies to our knowledge that have examined any effects caused by the expression of protease-dead separase in a wild-type background, which could reveal more information about the activity of this mutant protein.

In *C. elegans*, separase has been shown to regulate chromosome segregation [14], centriole duplication ([10], [15] and membrane trafficking [16]–[18]. However, the mechanism(s) by which separase controls these various cell division processes is not known. We created a transgenic worm line expressing protease-dead separase fused to GFP (SEP-1^PD^::GFP) using standard methods to characterize its expression in *C. elegans* embryos [17]. As previously reported, strains expressing SEP-1^PD^::GFP must be propagated on *gfp* RNAi, and removed from RNAi for several generations to examine expression [17]. In this report, we demonstrate that SEP-1^PD^::GFP expression causes embryo lethality. Other researchers have encountered similar difficulties with other mutant proteins [19], highlighting the need for methods to control transgene expression. Here, we methodologically characterize the usefulness of *gfp* RNAi as a way to propagate toxic transgenes in the *C. elegans* embryo.

We find that *gfp* RNAi silences SEP-1^PD^::GFP transgene expression and allows for maintenance of homozygous transgenic lines indefinitely. Upon removal from *gfp* RNAi, transgene re-expression takes several generations, with gradual reappearance of embryonic lethality. On average, we were able to propagate SEP-1^PD^::GFP worm lines for 5 generations after removal from *gfp* RNAi. SEP-1^PD^::GFP accumulates strongly at putative sites of separase activity, indicating that it could be substrate trapping. We also report that homozygous *sep-1* mutants expressing protease-dead separase are not viable.

We also describe a second method using male worms to propagate the transgene. The *pie-1* promoter is widely used to drive embryonic expression, and is expressed in the female germline [20]. Transgenic male worms carrying the SEP-1^PD^::GFP transgene can be crossed to *unc-119* hermaphrodites for many generations without obvious deleterious effects. The resulting hermaphrodites, carrying a single copy of SEP-1^PD^::GFP in a wild-type background, produce broods displaying high levels of embryonic lethality. Further, males can be used to propagate SEP-1^PD^::GFP reliably to facilitate transgene characterization and to combine transgene expression with mutant alleles. Using this strategy, we find that protease-dead separase exacerbates phenotypes in heterozygous mutants.

We have successfully used these newly developed methods to provide the first characterization of SEP-1^PD^::GFP in the *C. elegans* embryo. These methods employ standard laboratory techniques used by *C. elegans* researchers, which will open new possibilities for analysis of gene function in the *C. elegans* embryo. SEP-1^PD^::GFP expression causes embryonic lethality in WT animals, and does not rescue mutant separase animals. We conclude that protease-dead separase is dominant negative and interferes with endogenous separase function, a finding that was not reported in other systems. Collectively, our results suggest that protease-dead separase may trap substrates, as has been found for other catalytically inactivated enzymes [21], which would interfere with substrate cleavage by endogenous separase.

## Materials and Methods

### Strains

*C. elegans* strains were maintained according to standard protocols (Brenner, 1974). Temperature sensitive strains were maintained at 16°C, unless otherwise indicated in the text, and shifted to non-permissive temperature as indicated. All other strains were maintained at 20°C. Strains containing the protease-dead *sep-1* transgene were maintained on lawns of *gfp* RNAi feeding bacteria as indicated in text and below, then transferred onto OP50 lawns as indicated. A full list of strains used in this study and genotypes are included in Table 1.

**Table 1:** Strains used in this study.

Strain	Genotype	Reference and/or source
N2	Bristol (wild-type)	CGC
WH416	*unc-119(ed3)* III, *ojIs58[SEP-1::GFP unc119(+)]*	[16]
WH520	*unc-119(ed3)* III, *ojIs71[GFP::SEP-1(PD) unc119(+)]*	[17] and this study.
WH524	*unc-119(ed3)* III, *ojIs75[SEP-1(PD)::GFP unc119(+)]*	This study
WH408	*sep-1(e2406)* I/hT2*[bli-4(e937) let-? (q782) qls48]* I	[18]
VC1279	*sep-1(ok1749)* I/hT2 I	CGC
WH458	*sep-1(e2406)* I/hT2 I; *unc-119(ed3)* III/hT2 III, *ojIs58* [*GFP::SEP-1 unc119(+)*]	This study
WH548	*sep-1(e2406)* I/hT2 I; *unc-119(ed3)* III/hT2 III, *ojIs71* [*GFP::SEP-1(PD) unc119(+)*]	This study
WH504	*sep-1(ok1749)* I/hT2 I; *unc-119(ed3)* III/hT2 III, *ojIs58 [GFP::SEP-1 unc119(+)]*	This study
JAB7	*sep-1(ok1749)* I/hT2 I; *unc-119(ed3)* III/hT2 III, *ojIs71 [GFP::SEP-1(PD) unc119(+)]*	This study
WH488	*sep-1(e2406)* I/hT2 I; *unc-119(ed3)* III/hT2 III	This study
JAB3	*sep-1(ok1749)* I/hT2 I; *unc-119(ed3)* III/hT2 III	This study
RQ372	*unc-119(ed3)* III, *ojIs58[SEP-1::GFP unc119(+)]* itIs37 [*Ppie-1::mCherry::his-58 (pAA64) + unc-119(+)*] IV	Dr. Risa Kitagawa
JAB18	*unc-119(ed3)* III, *ojIs71[GFP::SEP-1(PD) unc119(+)]* itIs37 [*Ppie-1::mCherry::his-58 (pAA64) + unc-119(+)*]	This study

Some strains were obtained from the Caenorhabditis Genetics Center (CGC); see Table 1. Strain RQ372 was a kind gift from Dr. Risa Kitagawa. JAB18 was obtained by crossing WH520 males with OD56 hermaphrodites, and subsequent generations were maintained on *gfp* RNAi. At each generation following the cross, approximately half of the worms at L4 stage were moved to OP50 plates for 24 hours at 25°C and screened for the presence of both SEP-1^PD^::GFP and H2B::mCherry transgenes by microscopy. Worms were then singled from the original *gfp* RNAi feeding plate. This protocol was repeated until double homozygous transgenic lines were obtained, after which the line was maintained on *gfp* RNAi at 20°C. Feeding *gfp* RNAi did not silence expression of H2B::mCherry.

### Molecular Biology

**Figure 1:**
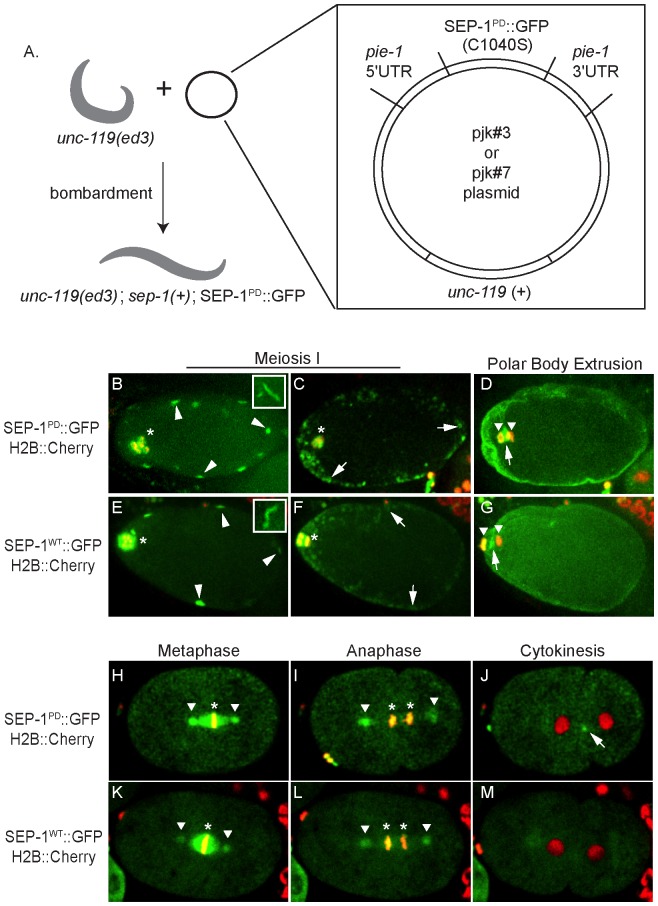
SEP-1^PD^::GFP transgenic worm lines. A. Microparticle bombardment of homozygous *unc-119(ed3); sep-1(+)* worms with plasmid DNA bound to gold beads. The plasmid (enlarged panel) contains the *sep-1* coding sequence with mutation in the protease domain (C1040S) fused to GFP under control of the *pie-1* promoter and an *unc-119(+)* rescue sequence, allowing for identification of transformed worm lines. The designated alleles and transgene are homozygous in the resulting transgenic worm line. B-M: SEP-1^PD^::GFP (top row) and SEP-1^WT^::GFP (bottom row) localization in newly fertilized embryos with H2B::mCherry. Embryos were imaged after 5 generations removed from *gfp* RNAi feeding (see text and Figure 2). During meiosis I, SEP-1^PD^::GFP and SEP-1^WT^::GFP localize to chromosomes and the meiotic spindle (asterisk, B,C, E, F). During prometaphase, separase appears on cortical filaments that appear as puncta depending on whether they are oriented parallel to the focal plane (arrowheads, B and E, insets show examples of filaments oriented properly). Separase is localized to cortical granules by the onset of anaphase (arrows, C and F). During polar body extrusion, SEP-1^PD^::GFP and SEP-1^WT^::GFP accumulate at the base of the polar body (base of the polar body designated by arrows D and G, respectively) between the separating anaphase chromosomes (chromosomes designated by arrowheads, D and G). SEP-1^PD^::GFP also accumulates strongly on the plasma membrane of the embryo after cortical granule exocytosis (D). During the indicated stages of mitosis (H-M), SEP-1^WT^::GFP and SEP-1^PD^::GFP localize to chromosomes (asterisk) and centrosomes (arrowhead). During cytokinesis, SEP-1^PD^::GFP accumulates at the cleavage furrow (arrow, J).

Cloning the protease-dead separase mutant DNA sequence into pjk#3 or pjk#7 vectors was performed as previously described [17]. Microparticle bombardment [22] was used to obtain transgenic worm lines as described in the text and Figure 1.

### RNAi feeding

The *gfp* RNAi feeding construct in L4440 vector was obtained from Dr. Scott Kennedy [23]. To silence GFP fusion transgenes and maintain worm lines, worms were picked onto lawns of *gfp* RNAi feeding bacteria and L4 worms were picked at each generation onto fresh lawns. In order to provide the optimal RNAi effect for transgene silencing, worms were grown on *gfp* RNAi at 20°C (which is the semi-permissive temperature for the *sep-1(e2406)* allele), as we were unable to propagate some lines on *gfp* RNAi by feeding at 16°C. For transgene re-expression, L4 worms were removed to OP50 lawns and picked onto fresh OP50 feeding plates at each generation as indicated in the text and figures.

### Microscopy

For imaging of mitotic embryos, young adult worms were dissected in M9 and embryos were mounted on agar pads as previously described [17]. For imaging of meiotic embryos, *in utero* imaging was performed using young adult worms immobilized in 1 mg/mL levamisole mounted on 2% agar pads and covered with a coverslip. Live cell imaging was performed using a Nikon Eclipse inverted microscope with a CSU-22 spinning disc imaging system equipped with a 60X 1.40NA objective from Visitech International, running metamorph software. Digital images were obtained with a Photometrics EM-CCD camera. Images were analyzed and time-lapse movies were made using FIJI (ImageJ) software using the Bio-Formats plugin from LOCI (www.loci.wisc.edu). Images were enhanced by adjusting minimum and maximum display levels in single color channels, then overlayed to display both channels.

## Results

### Creation of protease-dead separase transgenic worm lines

We created SEP-1^PD^::GFP expressing transgenic worm lines using microparticle bombardment, using standard protocols [22], but GFP expressing lines could not be maintained for more than a few generations. The final construct contains several features that allowed us to propagate lines carrying dominant negative transgenes. The construct is designed to generate proteins fused to GFP driven by the *pie-1* promoter (Figure 1A). Importantly, the *pie-1* promoter is widely used to drive transgene expression in *C. elegans* oocytes and young embryos [20]. The construct also has an *unc-119(+)* selection marker allowing for identification of transformed worms. We cloned genomic *sep-1* sequence, with a point mutation that results in cysteine to serine substitution at amino acid 1040, located in the protease domain of SEP-1. We created multiple independent worm lines with integrated transgenes coding for both N- and C-terminal fusions of GFP to SEP-1^PD^ using this strategy, all of which had severe growth defects.

We generated lines expressing both SEP-1^PD^::GFP and H2B::mCherry and examined localization of GFP tagged separase relative to chromosome segregation in the newly fertilized embryo (Figure 1B–M, Movies S1–S4). We found that SEP-1^PD^::GFP localizes similarly to SEP-1^WT^::GFP during meiosis I (Figure 1B-G, Movies S1 and S2) and mitosis (Figure 1H-M, Movies S3 and S4 and previously reported [17]). Both SEP-1^WT^::GFP and SEP-1^PD^::GFP localize to chromosomes and the meiotic spindle during meiosis (Figure 1B-C and E-F). SEP-1^WT^::GFP and SEP-1^PD^::GFP localize to filamentous structures (cortical filaments) in the oocyte prior to fertilization (not shown). We have previously reported the localization of separase and other proteins to cortical filaments [16], which are not well characterized. Following fertilization, during the progression of meiosis I, SEP-1^PD^::GFP moves from cortical filaments to cortical granules (Figure 1B-C, Movie S1) as does SEP-1^WT^::GFP (Figure 1E-F, Movie S2). Following cortical granule exocytosis and meiotic anaphase I, SEP-1^PD^::GFP associates strongly with the embryo plasma membrane for an extended period of time and with the base of the polar body as compared to SEP-1^WT^::GFP (Figure 1D and G, Movies S1 and S2). Interestingly, SEP-1^PD^::GFP accumulates strongly compared to SEP-1^WT^::GFP at several sites of putative action during mitosis, including centrosomes, mitotic spindle (compare Figure 1H-I and 1K-L) and the cleavage furrow during cytokinesis (compare Figure 1J and 1M, [17]). These sites of separase activity may contain substrates of separase, which may have stronger binding to the inactive protease leading to its accumulation relative to wild-type separase, suggesting that SEP-1^PD^::GFP could be substrate-trapping.

### Silencing of SEP-1^PD^::GFP expression by *gfp* RNAi

**Figure 2:**
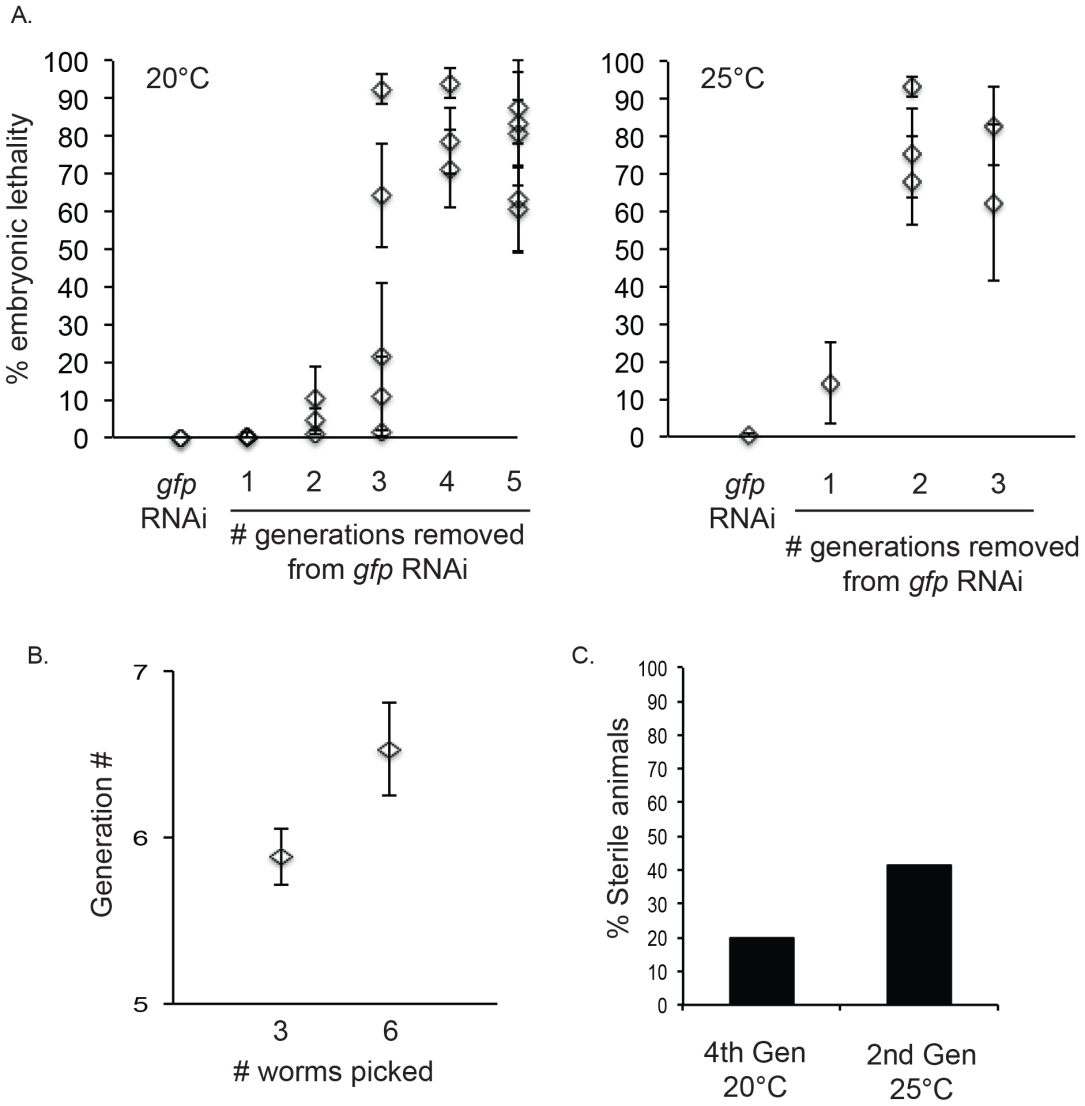
SEP-1^PD^::GFP worm lines can be maintained on *gfp* RNAi. A. Embryonic lethality of SEP-1^PD^::GFP line on *gfp* RNAi and after removal onto OP50 plates at 20°C (left) or 25°C (right). Each data point with error bars represents the average of embryonic lethality from 10 singled worms +/− SEM. B. Average generation +/− SEM that could be propagated for the SEP-1^PD^::GFP line after removal from *gfp* RNAi when the indicated number of worms are picked at each generation and kept at 20°C. C. Percentage of sterile animals in the SEP-1^PD^::GFP line after removal from *gfp* RNAi.

If protease-dead separase remains bound to substrates, it could interfere with their cleavage by endogenous separase, therefore having a dominant-negative effect. Consistent with a dominant negative activity, SEP-1^PD^::GFP expression caused embryo lethality (Figure 2 and Figure S1, see below) in the wild-type background with two copies of endogenous separase. Dominant negative activity of protease-dead separase has not been reported in other systems. High levels of embryonic lethality in SEP-1^PD^::GFP expressing worm lines required us to develop methods to propagate this “toxic” transgene for further examination. RNAi provides a reliable system for targeted gene knock down in *C. elegans*. We took advantage of RNAi in order to silence expression of the SEP-1^PD^::GFP transgene by maintaining transgenic worm lines on lawns of *gfp* RNAi feeding bacteria.

After bombardment following the standard protocol, Unc rescued animals were screened for GFP expression, and Unc rescued GFP positive lines had high lethality when grown under standard lab conditions. However, SEP-1^PD^::GFP transgenic worm lines fed *gfp* RNAi showed no embryonic lethality and could be propagated indefinitely at 20°C and 25°C (Figure 2A). When worms were transferred from *gfp* RNAi onto OP50, embryonic lethality returned after several generations (Figure 2A), and higher levels of embryonic lethality correlated with higher GFP expression levels. Interestingly, broods from individual worms showed similar levels of embryonic lethality within group at each generation after removal from RNAi (note error bars for each data point, Figure 2A), in contrast to variability in individual offspring from the same brood following injection of RNAi [24]. This difference could be the result of uniform RNAi administration when feeding RNAi continually over multiple generations and selective pressure that would favor animals with more effective RNAi response. The return of embryonic lethality was dependent on temperature, as embryonic lethality occurred in 3–5 generations at 20°C and 2–3 generations at 25°C, which could be due to reduced generational *gfp*(RNAi) transmission, increased transgene expression at 25°C, or an increase in cell cycle timing leading to a decrease in fidelity of division. Picking a larger number of worms at each generation allows for propagation of the transgenic line on OP50 through one more generation (Figure 2B). This could be due to effects with picking animals of different penetrance of generational RNAi propagation, as seen previously [24]. Similar results were obtained for multiple independent worm lines expressing both N-terminal and C-terminal SEP-1^PD^ GFP fusion proteins, indicating that the position of GFP fusion is not a factor (Figure S1). SEP-1^PD^ expressing worms that survive hatching show abnormal developmental phenotypes including tail defects, slow growth, and sterility (Figure 2C), suggesting that protease-dead separase interferes with normal developmental processes in addition to causing embryonic lethality. Because transgene expression is *pie-1* driven, and should be most highly expressed in the germline and deposited in the egg, these results suggest that SEP-1^PD^ expressing worms show phenotypes that are a result of defects in the developing embryo.

### Transgenic SEP-1^PD^::GFP males can be used to propagate dominant negative SEP-1^PD^::GFP to offspring

**Figure 3:**
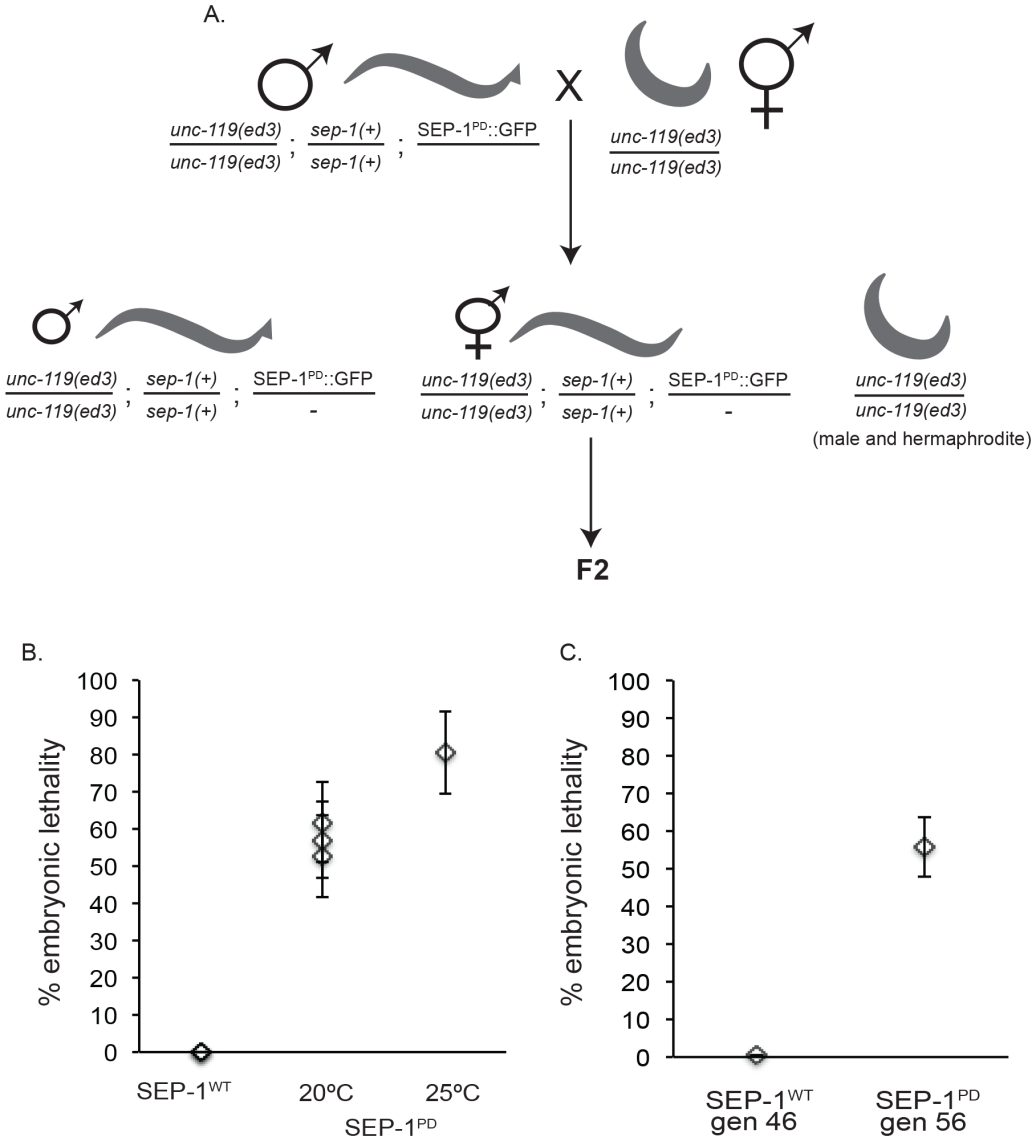
Propagation of the SEP-1^PD^::GFP transgene by backcrossing. A. The diagram represents the strategy used to propagate the SEP-1^PD^::GFP transgene using males. Transgenic *unc-119(ed3)/unc-119(ed3); sep-1(+)/sep-1(+)*; SEP-1^PD^::GFP/- males heterozygous for the transgene are continually crossed to *unc-119(ed3)* hermaphrodites to generate heterozygous *sep-1(+)/sep-1(+)*; SEP-1^PD^::GFP/- males and hermaphrodites in the *unc-119(ed3)* background. The resulting progeny (male and hermaphrodite) that carry the SEP-1^PD^::GFP transgene are readily identified by mobility because they are Unc rescued due to presence of the transgene. B. Embryonic lethality in F2 broods from singled F1 *sep-1(+)/sep-1(+)*; SEP-1^WT^::GFP/- or *sep-1(+)/sep-1(+)*; SEP-1^PD^::GFP/- hermaphrodites at the indicated temperature. C. Embryonic lethality in the F2 after the indicated number of backcrosses of heterozygous *sep-1(+)/sep-1(+)*; SEP-1^WT^::GFP/- or *sep-1(+)/sep-1(+)*; SEP-1^PD^::GFP/- transgenic males to *unc-119* hermaphrodites. Data points represent the average of a group of 10 singled worms +/− SEM.

Expression of most transgenes in the *C. elegans* embryo, including our SEP-1^PD^::GFP transgene, is under control of the maternal *pie-1* promoter [20]. We created transgenic SEP-1^PD^::GFP male worms by heat shock and backcrossed to *unc-119* hermaphrodites to easily identify Unc-rescued *sep-1(+)/sep-1(+)*; SEP-1^PD^::GFP/- males, which did not express significant levels of SEP-1^PD^::GFP and gave rise to many cross progeny for our studies (Figure S2). Typically, expression of *pie-1* driven transgenes is not observed in sperm, although expression in the male germline has been previously observed [25], which may depend where the transgene is integrated. We therefore reasoned that we could use males with a single copy of SEP-1^PD^::GFP to propagate the SEP-1^PD^::GFP transgene. From the F1 progeny, males heterozygous for the transgene (in *unc-119(ed3); sep-1(+)* homozygous background) crossed to *unc-119(ed3)* homozygous hermaphrodites produce heterozygous SEP-1^PD^::GFP males (Unc rescued), heterozygous SEP-1^PD^::GFP hermaphrodites (Unc rescued), and both male and hermaphrodite *unc-119* offspring (Figure 3A). The heterozygous SEP-1^PD^::GFP hermaphrodites, identified by Unc rescue (mobility), can be used for analysis of transgene expression, while the transgenic heterozygous males (also identified by Unc rescue) were continually backcrossed to *unc-119* hermaphrodites to maintain the line (Figure 3A), and were also used in crosses with other worm lines to test for genetic interactions. Single F1 heterozygous transgenic SEP-1^PD^::GFP hermaphrodites were picked onto individual plates and their progeny were analyzed for embryonic lethality. We found that embryonic lethality in the F2 brood was consistently in the range of 40–60% (Figure 3B), which shows that even a single copy of SEP-1^PD^::GFP has dominant-negative effects in a wild-type background. After backcrossing males to *unc-119* hermaphrodites more than 50 generations, embryonic lethality in the F2 remained within this range (Figure 3C), regardless of C-terminal or N-terminal GFP fusion (not shown). Propagation of SEP-1^PD^::GFP in males bypasses lethality and can be done indefinitely, while also providing consistent transgene expression in the F1 generation. Therefore, this approach provides a convenient strategy to introduce transgenes into different mutant backgrounds to test for genetic interactions (see below) or to create worm lines in combination with other transgenes.

### Genetic interactions of protease-dead separase with separase mutants

Previous studies demonstrated that protease-dead separase can rescue some loss of function separase phenotypes [11]–[13]. Therefore, we examined genetic interactions of SEP-1^PD^::GFP with mutant separase alleles: the hypomorphic *sep-1(e2406)* allele and the *sep-1(ok1749)* deletion allele. The *sep-1(e2406)* homozygous mutant is temperature sensitive and viable at 16°C, but 100% embryo lethal at the semi-permissive temperature, 20°C. The *sep-1(ok1749)* deletion mutant is likely a null allele since no protein can be detected by western blot [18]. At all temperatures, nearly all homozygous *sep-1(ok1749)* progeny die during embryogenesis, with very few surviving animals that arrest at early larval stages. Both *sep-1(e2406)* and *sep-1(ok1749)* are maintained as balanced heterozygotes with the *hT2[bli-4(e937) let-? (q782) qls48]* balancer chromosome which encodes GFP localized to the pharynx (hT2g). hT2g is a translocation balancer that can be used to balance mutations in LGI or LGIII. Scoring for GFP expression in the pharynx allows for identification of heterozygous (GFP+ pharynx) and homozygous (GFP- pharynx) mutants.

We examined *sep-1* mutant embryos expressing SEP-1^WT^::GFP or SEP-1^PD^::GFP, to determine if either of these transgenes can rescue *sep-1* mutants. We generated lines that were homozygous for either the SEP-1^WT^::GFP or SEP-1^PD^::GFP transgenes in these balanced separase mutant backgrounds. Both the balanced heterozygous *sep-1(e2406)* and *sep-1(ok1749)* deletion mutants with the SEP-1^PD^::GFP transgene could not be maintained on normal OP50 bacterial plates. Therefore, the balanced separase mutant lines with SEP-1^PD^::GFP were maintained on *gfp* RNAi at 20°C, because *gfp* RNAi feeding at 16°C did not allow for propagation of the strains (not shown). Furthermore, the balanced separase mutant lines homozygous for SEP-1^PD^::GFP could only be propagated for a maximum of 1-3 generations off of *gfp* RNAi at 20°C (compared to an average of 5 generations in the *sep-1(+)/sep-1(+)* background, Figure 2) before all progeny died, arrested prior to reaching adult, or were completely sterile.

**Figure 4:**
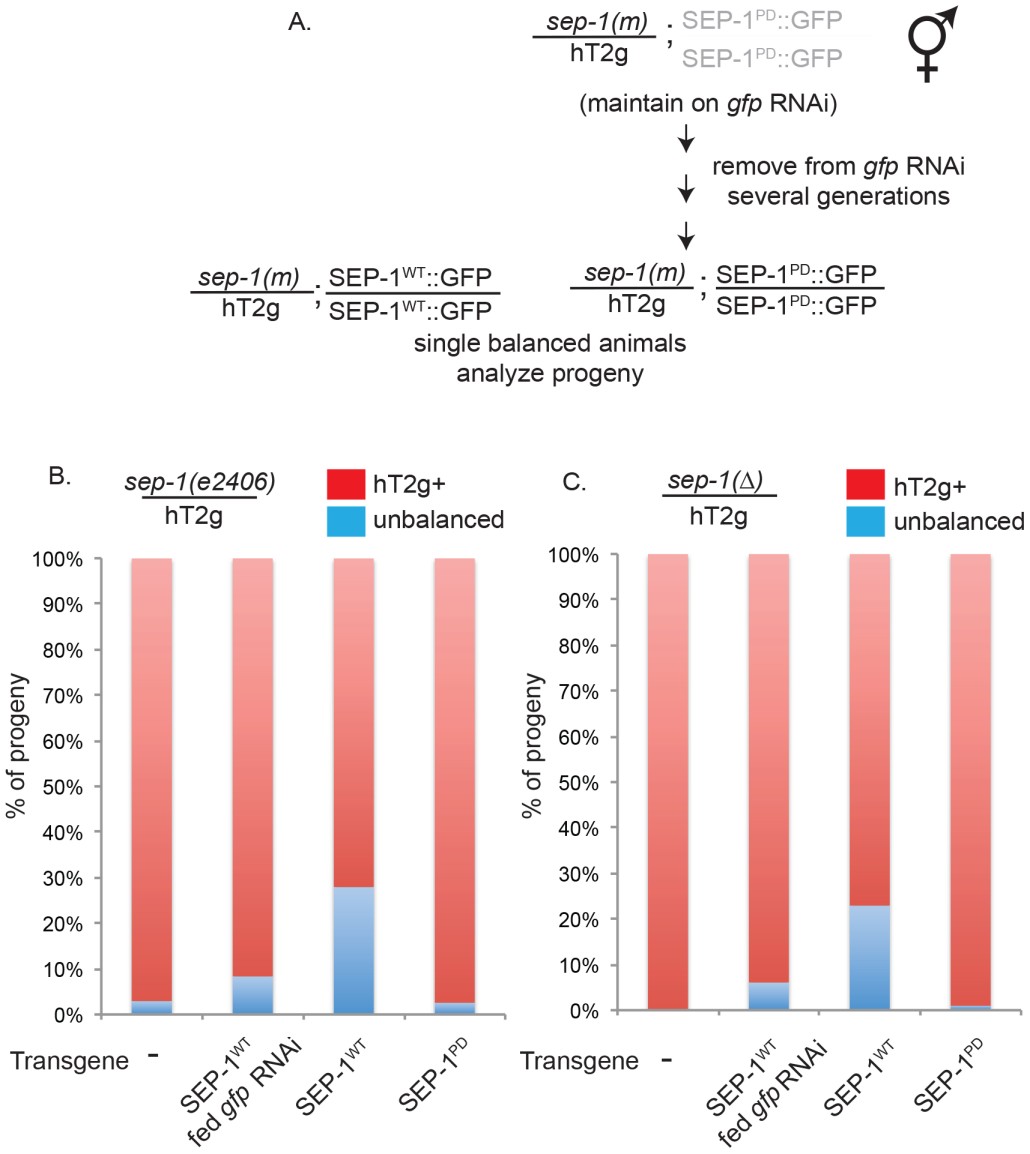
Wild-type SEP-1, but not protease-dead SEP-1, rescues *sep-1* mutants. A. Lines with SEP-1^PD^::GFP transgene were maintained on *gfp* RNAi at 20°C and removed for several generations to allow transgene expression. Balanced worms were then singled and progeny were analyzed. B. Percentage of progeny that are balanced mutant or homozygous mutant from singled *sep-1(e2406)*/hT2g hermaphrodites homozygous for the indicated transgene. C. Percentage of progeny that are balanced mutant or homozygous mutant from singled *sep-1(ok1749)*/hT2g (referred to as Δ) hermaphrodites with indicated transgene at 20°C.

We analyzed lethality in embryos from heterozygous, balanced mutant animals homozygous for SEP-1^WT^::GFP or SEP-1^PD^::GFP transgenes (Figure 4A). Consistent with the growth defects we observed, embryonic lethality in balanced mutant lines homozygous for SEP-1^PD^::GFP was more severe than the mutant alone, and this effect was reduced when transgene expression was silenced with *gfp* RNAi (not shown). Further, SEP-1^WT^::GFP expression was able to rescue both homozygous *sep-1(e2406)* and *sep-1(ok1749)* mutant progeny while SEP-1^PD^::GFP could not (Figure 4B and 4C). Importantly, expression of SEP-1^WT^::GFP can rescue both homozygous *sep-1(e2406)* hypomorphic and *sep-1(ok1749)* deletion mutants to produce a few gravid adult animals (not shown). These data indicate that protease-dead separase does not rescue viability in separase mutant embryos.

**Figure 5:**
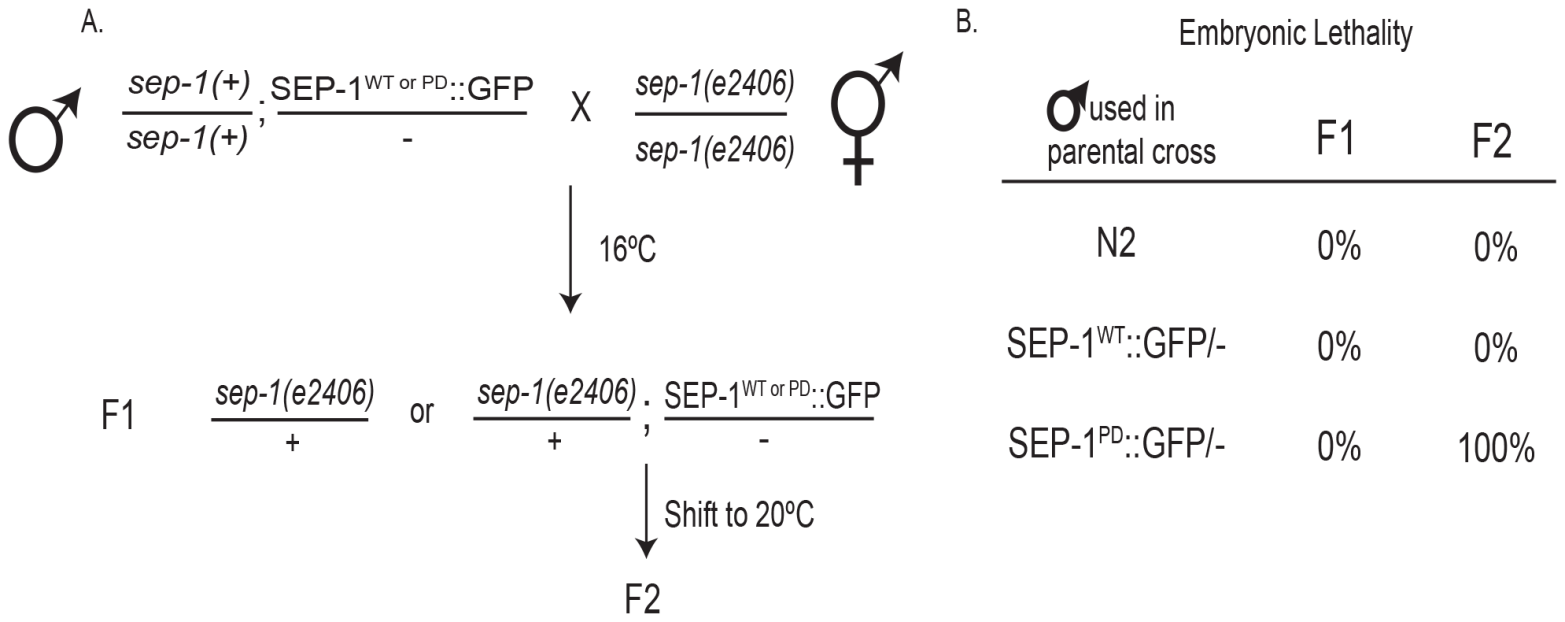
Genetic interactions of wild-type or protease-dead SEP-1 with *sep-1(e2406)*. A. Crossing scheme of heterozygous SEP-1^WT^::GFP or SEP-1^PD^::GFP transgenic males to *sep-1(e2406)* homozygous hermaphrodites. GFP transgene expression in the F1 was determined by microscopy after the shift to 20°C. Only progeny from animals expressing SEP-1^WT^::GFP or SEP-1^PD^::GFP were analyzed. B. The table shows embryonic lethality in the F1 and F2 progeny when males carrying the indicated transgene were used in the initial cross.

We employed our transgenic male propagation method to examine the phenotype of *sep-1(e2406)*/+ embryos expressing SEP-1^WT^::GFP or SEP-1^PD^::GFP. Transgenic SEP-1^WT^::GFP or SEP-1^PD^::GFP males were crossed with *sep-1(e2406)* homozygous hermaphrodites at 16°C, and F1 progeny were grown to L4 at 16°C (Figure 5A). Unfortunately all other mutant alleles of separase, including *sep-1(ok1749)*, are not viable as homozygotes and could not be tested this way. We attempted to cross *sep-1(ok1749)*/hT2g and *sep-1(e2406)*/hTg, both with the *unc-119(ed3)* background, hermaphrodites to transgenic SEP-1::GFP males to obtain the desired genotype, but had results inconsistent with the expected outcome in the F2 generation (not shown). Briefly, we observed that the SEP-1^PD^::GFP transgene was not expressed in the Unc rescued F2 as expected, although the SEP-1^WT^::GFP transgene was expressed. Given that the hT2g balancer breakdown has previously been reported [26], it is possible that there was a potential issue with hT2g. The F1 *sep-1(e2406)*/+; SEP-1^WT^::GFP/- and *sep-1(e2406)*/+; SEP-1^PD^::GFP/- worms were shifted to 20°C at the L4 stage and embryonic lethality was determined in the F2 brood. GFP expression was confirmed in the oocytes and embryos of the F1 hermaphrodites used in this analysis that give rise to F2 broods. Since the oocyte and early embryo is determined by the maternal genotype due to maternal deposition of cellular machinery, early F2 embryos reflect the *sep-1(e2406)*/+; SEP-1^WT or PD^::GFP maternal genotype. F2 embryos from *sep-1(e2406)*/+; SEP-1^WT^::GFP/- animals were fully viable as expected, but *sep-1(e2406)*/+; SEP-1^PD^::GFP/- F2 progeny showed 100% embryonic lethality (Figure 5B). This is consistent with a dominant negative activity of SEP-1^PD^::GFP, because SEP-1^PD^::GFP causes lethality in separase wild-type background (Figures 2 and 3), and enhances the phenotype of mutant separase alleles (Figure 4 and 5).

## Discussion

We employed the methods described in this manuscript to examine the consequence of SEP-1^PD^ expression in the *C. elegans* embryo. We find that protease-dead separase is dominant negative and likely interferes with endogenous separase function in our system. A dominant negative activity of protease-dead separase has not been reported in other systems, and could alter the interpretation of phenotypes of cellular expression of protease-dead separase. If dominant negative activity of protease-dead separase arises due to substrate trapping by the mutant separase enzyme, substrate cleavage by endogenous separase could be prevented and cause embryo lethality. In support of this, we find accumulation of protease-dead separase at putative sites of separase activity where it could be preventing access to substrates by endogenous separase. However, protease-dead separase could still potentially rescue non-proteolytic functions of separase. Further, expression of protease-dead separase in mutant cells that are not true null alleles can further confound interpretations. This could explain discrepancies in findings and conclusions regarding the protease function of separase in anaphase spindle elongation. Using temperature sensitive separase alleles in budding yeast, two groups [27], [28] concluded that separase proteolytic function was not required for anaphase spindle elongation. However, using more stringent alleles, [29] and [7] came to the opposite conclusion.

The lack of an inducible gene expression system in *C. elegans* makes it difficult to study dominant-negative or otherwise toxic mutant proteins. Here we discuss methods that facilitate studies involving worm lines with toxic *pie-1* driven transgenes. Although an inducible heat shock promoter has been suggested for inducing transgene expression [30], this approach does not lead to germline specific expression which could complicate phenotypic analysis. Combining soma or germline specific RNAi mutants (for example, *rrf-1* vs. *ppw-1* mutants) with *gfp* RNAi and inducible expression might be a way to circumvent this problem. While we were able to create SEP-1^PD^::GFP transgenic lines by normal methods, recovery of bombarded animals directly on *gfp* RNAi could allow isolation of worm lines with transgenes that are more toxic than SEP-1^PD^ or allow for the isolation of overexpressing lines. These methods could also be combined with mutants defective in generational RNAi or temperature sensitive mutations in the RNAi machinery to more rapidly shut off the multigenerational RNAi silencing mechanism and more quickly induce transgene expression [31], [32]. In addition, bombardment of *him;unc* lines could allow for immediate isolation of transgenic males, which can be maintained by backcrossing. The male propagation method bypasses the multigenerational propagation of *gfp* RNAi, but only introduces a single copy of the transgene, which may not lead to highest expression levels. On the other hand, backcrossing to *unc-119* each generation can help reduce selective pressure that might silence transgene expression or select for suppressor mutations.

The *gfp* RNAi feeding and male propagation methods allow for crossing schemes to study genetic interactions of mutant proteins. Mutant separase alleles are lethal when homozygous and must be maintained as heterozygotes with chromosomal balancers. We found that SEP-1^PD^::GFP could not rescue hypomorphic or null separase mutants, and that SEP-1^PD^::GFP expression exacerbated phenotypes in heterozygous hypomorphic separase mutants. We were unable to examine SEP-1^PD^::GFP in separase null background because separase null worms are not viable. Further, male propagation and *gfp* RNAi feeding allow for the creation of double transgenic lines to study localization patterns and phenotypes by live imaging.

With the current advancements of CRISPR-Cas genome editing [33], the use of *gfp* RNAi and similar strategies may be advantageous. For example, *gfp* RNAi could be used to knock down expression of any endogenous gene of interest tagged with GFP. This strategy may also prove more effective than gene-specific RNAi for RNAi resistant genes. Further, one could temporally control mutant allele expression for multiple alleles by designing and utilizing *gfp* RNAi in combination with another, such as RNAi directed against mCherry.

Further analysis is required to determine which functions of separase require protease activity in the *C. elegans* embryo. Previous work indicated that polar body extrusion is independent of separase's proteolytic activity in mouse oocytes [6]. However, this analysis was performed in separase-null mouse embryos, which may have a different phenotype than seen in wild-type separase background. For example, separase could require autocleavage to efficiently bind substrates, which could be mediated in our SEP-1^PD^ transgenic lines by endogenous separase. Ultimately, detailed mechanistic understanding of separase function will require the identification of relevant substrates and characterization of how their cleavage works together with non-proteolytic signaling mechanisms to execute various cell division events.

## Supporting Information

Figure S1 **Embryonic lethality in lines with N-terminal or C-terminal GFP fusion to SEP-1^PD^.** Both WH520 (C-terminal fusion to SEP-1^PD^) and WH524 (N-terminal fusion to SEP-1^PD^) could be maintained on *gfp* RNAi (not shown). The graph shows embryonic lethality for WH520 and WH524 following removal from *gfp* RNAi at 20°C for the indicated number of generations. Each data point with error bars represents the average of a group of 10 singled worms +/- SEM examined in an individual experiment.
(PDF)

Figure S2 **GFP expression in the male germline.** GFP expression in N2 males (A) and WH520 males (B). Regions corresponding to sperm (box with dashed line) and testes (box with solid line) are outlined. C. Image of gonad of an F1 SEP-1^PD^::GFP hermaphrodite derived from the cross outlined in Figure 3. The -1 oocyte and +1 and +2 embryos are designated by labels and the spermatheca is outlined by the box with dashed line.
(PDF)

Movie S1 **Meiosis I, including cortical granule exocytosis and the first polar body extrusion, in an embryo expressing SEP-1^PD^::GFP and H2B::mCherry.** The movie shows a maximum projection of selected 1 µm z stacks to display cortical granules and chromosomes. Images were acquired every 20 seconds. Playback rate of the movie is 10 frames per second.
(AVI)

Movie S2 **Meiosis I, including cortical granule exocytosis and the first polar body extrusion, in an embryo expressing SEP-1^WT^::GFP and H2B::mCherry.** The movie shows a maximum projection of selected 1 µm z stacks to display cortical granules and chromosomes. Images were acquired every 20 seconds. Playback rate of the movie is 10 frames per second.
(AVI)

Movie S3 **Mitosis in an embryo expressing SEP-1^PD^::GFP and H2B::mCherry.** Images of a single z plane were acquired every 30 seconds. Playback rate of the movie is 10 frames per second.
(AVI)

Movie S4 **Mitosis in an embryo expressing SEP-1^WT^::GFP and H2B::mCherry.** Images of a single z plane were acquired every 30 seconds. Playback rate of the movie is 10 frames per second.
(AVI)
