# Supplementary material for: Protease-Dead Separase Is Dominant Negative in the C. elegans Embryo
Source: PLoS One. 2014 Sep 22;9(9):e108188. doi: 10.1371/journal.pone.0108188 (PMC4171520; doi:10.1371/journal.pone.0108188)
Supplement: Figure S1 — Embryonic lethality in lines with N-terminal or C-terminal GFP fusion to SEP-1PD. Both WH520 (C-terminal fusion to SEP-1PD) and WH524 (N-terminal fusion to SEP-1PD) could be maintained on gfp RNAi (not shown). The graph shows embryonic lethality for WH520 and WH524 following removal from gfp RNAi at 20°C for the indicated number of generations. Each data point with error bars represents the average of a group of 10 singled worms +/- SEM examined in an individual experiment. (PDF) [file pone.0108188.s001.pdf]

SUPPLEMENTAL FIGURE 1

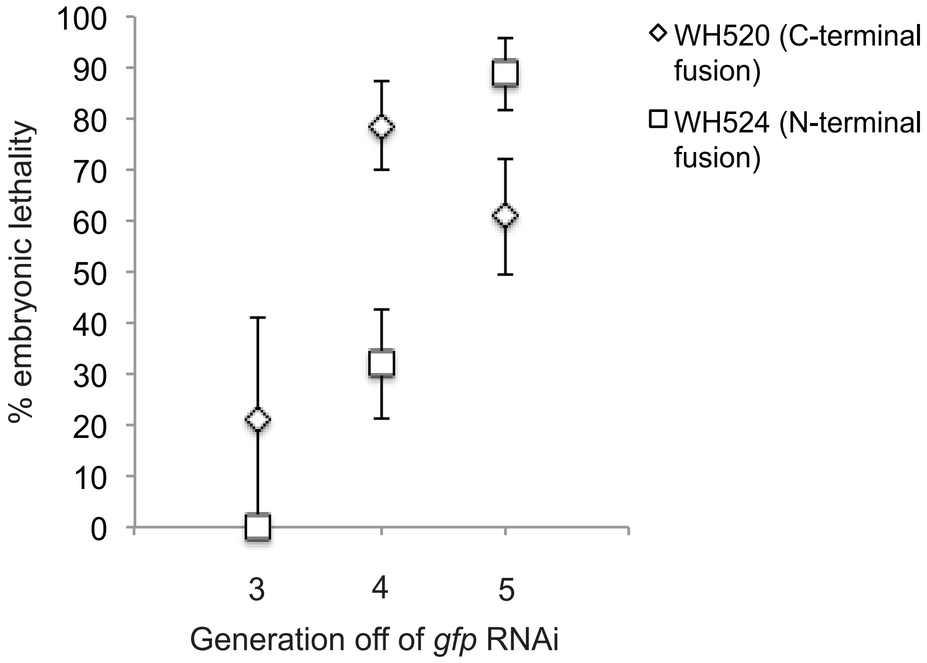

Supplemental Figure 1. Embryonic lethality in lines with N-terminal or C-terminal GFP fusion to SEP-1PD. Both WH520 (C-terminal GFP fusion to SEP-1PD) and WH524 (N-terminal GFP fusion to SEP-1PD) could be maintained on gfp RNAi (not shown). The graph shows embryonic lethality for WH520 and WH524 following removal from gfp RNAi at 20°C for the indicated number of generations. Each data point with error bars represents the average of a group of 10 singled worms +/- SEM examined in an individual experiment.
