# Supplementary material for: Protease-Dead Separase Is Dominant Negative in the C. elegans Embryo
Source: PLoS One. 2014 Sep 22;9(9):e108188. doi: 10.1371/journal.pone.0108188 (PMC4171520; doi:10.1371/journal.pone.0108188)
Supplement: Figure S2 — GFP expression in the male germline. GFP expression in N2 males (A) and WH520 males (B). Regions corresponding to sperm (box with dashed line) and testes (box with solid line) are outlined. C. Image of gonad of an F1 SEP-1PD::GFP hermaphrodite derived from the cross outlined in Figure 3. The -1 oocyte and +1 and +2 embryos are designated by labels and the spermatheca is outlined by the box with dashed line. (PDF) [file pone.0108188.s002.pdf]

SUPPLEMENTAL FIGURE 2

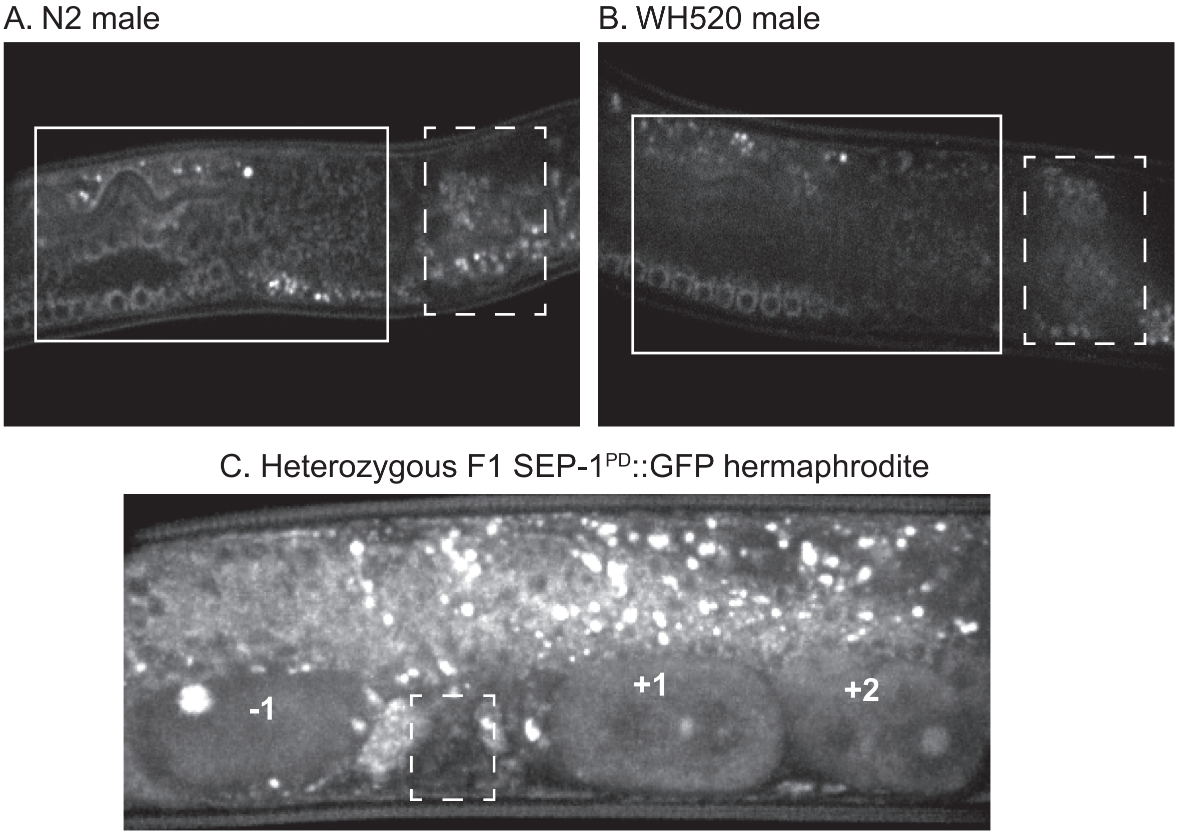

Supplemental Figure 2. GFP expression in the male germline. GFP expression in N2 males (A) and WH520 males (B). Regions corresponding to sperm (box with dashed line) and testes (box with solid line) are outlined. C. Image of gonad of an F1 SEP-1^{PD}::GFP hermaphrodite derived from the cross outlined in Figure 3. The -1 oocyte and +1 and +2 embryos are designated by labels and the spermatheca is outlined by the box with dashed line.
